# Common mtDNA variations at C5178a and A249d/T6392C/G10310A decrease the risk of severe COVID-19 in a Han Chinese population from Central China

**DOI:** 10.1186/s40779-021-00351-2

**Published:** 2021-11-01

**Authors:** Yi Wu, Xian-Hui Wang, Xi-Hua Li, Li-Yuan Song, Shi-Long Yu, Zhi-Cheng Fang, Yu-Quan Liu, Le-Yong Yuan, Chun-Yan Peng, Shen-Yi Zhang, Wang Cheng, Hong-Chao Ma, Li-Feng Wang, Jun-Ming Tang, Yun-Fu Wang, Fu-Yun Ji

**Affiliations:** 1 Department of Medical Biology, School of Basic Medical Science, Hubei University of Medicine, Shiyan, 442000 Hubei China; 2 Institute of Biomedical Research, Hubei University of Medicine, Shiyan, 442000 Hubei China; 3 Institute of Human Respiratory Disease, Xinqiao Hospital, The Army Medical University (Third Military Medical University), 400037 Chongqing, China; 4 Department of Emergency Medicine, Taihe Hospital, Hubei University of Medicine, Shiyan, 442000 Hubei China; 5 Department of Geriatric Medicine, Taihe Hospital, Hubei University of Medicine, Shiyan, 442000 Hubei China; 6 Department of Immunology, School of Basic Medical Sciences, Hubei University of Medicine, Shiyan, 442000 Hubei China; 7 Department of Laboratory Medicine, Taihe Hospital, Hubei University of Medicine, Shiyan, 442000 Hubei China; 8 Hubei Key Laboratory of Embryonic Stem Cell Research, School of Basic Medical Science, Hubei University of Medicine, Shiyan, 442000 Hubei China; 9 Department of Neurology, Taihe Hospital, Hubei University of Medicine, Shiyan, 442000 Hubei China

**Keywords:** MtDNA variations, SARS-CoV-2, COVID-19, Risk, Han Chinese

## Abstract

**Background:**

Mitochondria have been shown to play vital roles during severe acute respiratory syndrome coronavirus 2 (SARS-CoV-2) infection and coronavirus disease 2019 (COVID-19) development. Currently, it is unclear whether mitochondrial DNA (mtDNA) variants, which define mtDNA haplogroups and determine oxidative phosphorylation performance and reactive oxygen species production, are associated with COVID-19 risk.

**Methods:**

A population-based case–control study was conducted to compare the distribution of mtDNA variations defining mtDNA haplogroups between healthy controls (*n* = 615) and COVID-19 patients (*n* = 536). COVID-19 patients were diagnosed based on molecular diagnostics of the viral genome by qPCR and chest X-ray or computed tomography scanning. The exclusion criteria for the healthy controls were any history of disease in the month preceding the study assessment. MtDNA variants defining mtDNA haplogroups were identified by PCR-RFLPs and HVS-I sequencing and determined based on mtDNA phylogenetic analysis using Mitomap Phylogeny. Student’s *t*-test was used for continuous variables, and Pearson’s chi-squared test or Fisher’s exact test was used for categorical variables. To assess the independent effect of each mtDNA variant defining mtDNA haplogroups, multivariate logistic regression analyses were performed to calculate the odds ratios (*OR*s) and 95% confidence intervals (CIs) with adjustments for possible confounding factors of age, sex, smoking and diseases (including cardiopulmonary diseases, diabetes, obesity and hypertension) as determined through clinical and radiographic examinations.

**Results:**

Multivariate logistic regression analyses revealed that the most common investigated mtDNA variations (> 10% in the control population) at C5178a (in NADH dehydrogenase subunit 2 gene, *ND2*) and A249d (in the displacement loop region, D-loop)/T6392C (in cytochrome c oxidase I gene, *CO1*)/G10310A (in *ND3*) were associated with a reduced risk of severe COVID-19 (*OR* = 0.590, 95% CI 0.428–0.814, *P* = 0.001; and *OR* = 0.654, 95% CI 0.457–0.936, *P* = 0.020, respectively), while A4833G (*ND2*), A4715G (*ND2*), T3394C (*ND1*) and G5417A (*ND2*)/C16257a (D-loop)/C16261T (D-loop) were related to an increased risk of severe COVID-19 (*OR* = 2.336, 95% CI 1.179–4.608, *P* = 0.015; *OR* = 2.033, 95% CI 1.242–3.322, *P* = 0.005; *OR* = 3.040, 95% CI 1.522–6.061, *P* = 0.002; and *OR* = 2.890, 95% CI 1.199–6.993, *P* = 0.018, respectively).

**Conclusions:**

This is the first study to explore the association of mtDNA variants with individual’s risk of developing severe COVID-19. Based on the case–control study, we concluded that the common mtDNA variants at C5178a and A249d/T6392C/G10310A might contribute to an individual’s resistance to developing severe COVID-19, whereas A4833G, A4715G, T3394C and G5417A/C16257a/C16261T might increase an individual’s risk of developing severe COVID-19.

**Supplementary Information:**

The online version contains supplementary material available at 10.1186/s40779-021-00351-2.

## Background

The coronavirus disease 2019 (COVID-19) pandemic caused by severe acute respiratory syndrome coronavirus 2 (SARS-CoV-2) has resulted in a worldwide crisis of formidable morbidity and mortality. The epidemiology, diagnosis, risk factors and treatments of COVID-19 have been explored intensively since the outbreak in Wuhan (Hubei Province, China) in December 2019. Many studies have shown that the clinical features of COVID-19 range from an asymptomatic state to acute respiratory distress syndrome (ARDS) and multiorgan dysfunction. Most COVID-19 patients develop a respiratory tract infection with common symptoms of cough, fever and shortness of breath. Other reported symptoms are weakness, malaise, respiratory distress, muscle pain, sore throat and loss of taste and/or smell. A number of patients develop severe fatal consequences resulting from a surge of inflammatory events (also known as the cytokine storm) [1, 2]. These clinical characteristics hint that although SARS-CoV-2 infection is the causative factor of COVID-19, not all individuals exposed to SARS-CoV-2 will develop COVID-19, especially severe COVID-19, strongly suggesting that the gene-environment interactions exist in COVID-19 progression. An individual’s hereditary susceptibility and innate capacities of antioxidant and immune responses to SARS-CoV-2 might contribute to this process. Currently, known factors related to an individual’s susceptibility to severe COVID-19 include: advanced age; male sex; blood group A in Europeans; comorbidities of cardiopulmonary diseases, diabetes, obesity and hypertension [3]; a genomic segment of ~ 50 kb inherited from Neanderthals currently carried by ~ 50% of people in South Asia and ~ 16% of people in Europe [4]; a 3p21.31 gene cluster [5]; mutations in 13 protein-coding genes of the interferon pathway [6]; and other loci [7]. Whether there are other specific molecular markers to predict the risk of COVID-19 remains unclear.

Recently, mitochondria have been inferred to be interrelated and interacted with oxidative stress and inflammation during SARS-CoV-2 infection and COVID-19 progression [8, 9]. Furthermore, mitochondria have been shown to be indispensable regulators of innate and adaptive immune responses [10] and the activation, development, maintenance and survival of immune cells [11]. These findings provide clues that mitochondria might be associated with an individual’s susceptibility to COVID-19.

As the hub of cellular oxidative homeostasis, mitochondria generate approximately 85% reactive oxygen species (ROS) when they produce usable energy through oxidative phosphorylation (OXPHOS). In contrast to other cellular organelles, mitochondria have their own DNA (mtDNA). Interestingly, the common “nonpathological” mtDNA variation defining mtDNA haplogroups determines OXPHOS performance and ROS production in humans and mice [12]. Additionally, these mtDNA variations exert a considerable influence on longevity [13], help human beings adapt to different environments [14, 15], and are associated with susceptibility to human diseases in conditions where ROS generated by mitochondria play a part [16–19]. Biological functional analysis by introducing human-associated point mutations into yeast mtDNA has revealed that certain human mtDNA variations in the key catalytic domains of mtDNA-CYB significantly changed the complex III activity or drug sensitivity of yeast [20]. Here, we hypothesized that certain mtDNA variants defining mtDNA haplogroups might be related to an individual’s susceptibility to COVID-19. To test this hypothesis, we performed a population-based case–control study for the first time to compare the distribution of mtDNA variants defining mtDNA haplogroups between COVID-19 patients and healthy controls in a Han Chinese population from Central China.

## Methods

### Study population

This population-based case–control study was approved by the Ethics Committee of Hubei University of Medicine (Hubei, China) (2020-TH-063 and 2020-TH-064). COVID-19 patients (*n* = 536) were recruited from Taihe Hospital (the First Affiliated Hospital of Hubei University of Medicine, Shiyan, Hubei Province, China) and the People’s Hospital of Hubei Province (the Affiliated Hospital of Wuhan University, Wuhan, Hubei Province, China) from February 2020 to March 2020. COVID-19 patients were diagnosed based on molecular diagnostics of the viral genome by qPCR and chest X-ray or computed tomography scanning, and stratified into cases with moderate (non-ICU) and severe (ICU) disease (patients with ARDS, multiple organ dysfunction, or metabolic acidosis). Age- and sex-matched healthy volunteers (*n* = 615) were individuals who underwent physical examinations at the two hospitals. The exclusion criterion for the healthy controls was any history of disease in the one-month preceding the study assessment. All subjects were unrelated for at least three generations. After explaining the purpose and procedures of the study, all the participants signed a written informed consent form and completed a detailed questionnaire on their smoking habits.

### Genomic DNA extraction

Three millilitres of peripheral blood from each subject were drawn into Na-EDTA tubes. After incubation at 55 °C for 30 min to inactivate the potential SARS-CoV-2, the blood samples were stored at -80 °C prior to genomic DNA extraction. Genomic DNA was extracted from peripheral blood using the Ezup Column Blood Genomic DNA Purification Kit (Lot#: B518253-0100, Sangon Biotech Co., Ltd, Shanghai, China).

### Detection of mtDNA variations defining mtDNA haplogroups

MtDNA variants defining mtDNA haplogroups were identified using PCR-restriction fragment length polymorphism (PCR–RFLP) and replenished by hypervariable segment I (HVS-I) sequencing as previously described [15, 16, 18, 19]. Briefly, after the entire mtDNA was amplified into 22 overlapping PCR fragments, the PCR fragments were digested with different restriction endonucleases and replenished by sequencing HVS-I. Two × Taq Plus Master Mix II was used for PCR–RFLP and HVS-I sequencing (Lot#: P213-01, Vazyme Co., Ltd, Nanjing, China). The restriction endonucleases *Alu*I, *Ava*II, *Bam*HI, *Bst*NI, *Dde*I, *Hae*II, *Hae*III, *Hha*I, *Hinc*II and *Hinf*I were used in this study (Takara Co., Ltd, Dalian, China). The primers for PCR-RFLPs and HVS-I sequencing were synthesized by Sangon Biotech Co., Ltd. (Shanghai, China), and the primer sequences are presented in Additional file 1: Table S1. The mtDNA polymorphisms defining mtDNA haplogroups were determined based on mtDNA phylogenetic analysis using Mitomap Phylogeny [14]. MtDNA variants were given in the format [ancestral base][position number][derived base]. The Human Genome Variation Society (HGVS) validation of the mtDNA variants is presented in Additional file 1: Table S2.

### Comparison of the mtDNA variants defining mtDNA haplogroups between cases and controls

To explore whether the mtDNA variants defining mtDNA haplogroups were associated with individual’s susceptibility to COVID-19, the distribution of mtDNA variants defining mtDNA haplogroups were compared between the pooled cases and controls after the mtDNA variants defining mtDNA haplogroups were identified using PCR–RFLP replenished by HVS-I sequencing for all of the subjects. Additionally, the distribution of mtDNA variants defining mtDNA haplogroups were analysed between controls and moderate cases or severe cases.

### Data analysis

Student’s *t*-test was used for continuous variables, and Pearson’s chi-squared test or Fisher’s exact test was used for categorical variables. For multiple comparisons of mtDNA variants defining mtDNA haplogroups, Bonferroni correction was applied (the required significance level was *P* = 0.05/number of comparisons). To assess the independent effect of each mtDNA variant defining mtDNA haplogroups, multivariate logistic regression analyses were performed to calculate the odds ratios (*OR*s) and 95% confidence intervals (CIs) with adjustments for the possible confounding factors of age, sex, smoking and diseases (including cardiopulmonary diseases, diabetes, obesity and hypertension) as determined through clinical and radiographic examinations. All statistical analyses were performed using SPSS Statistics 25 for Mac (SPSS Inc., Chicago, IL, USA).

## Results

### MtDNA variations defining mtDNA haplogroups in controls and pooled cases

**Fig. 1:**
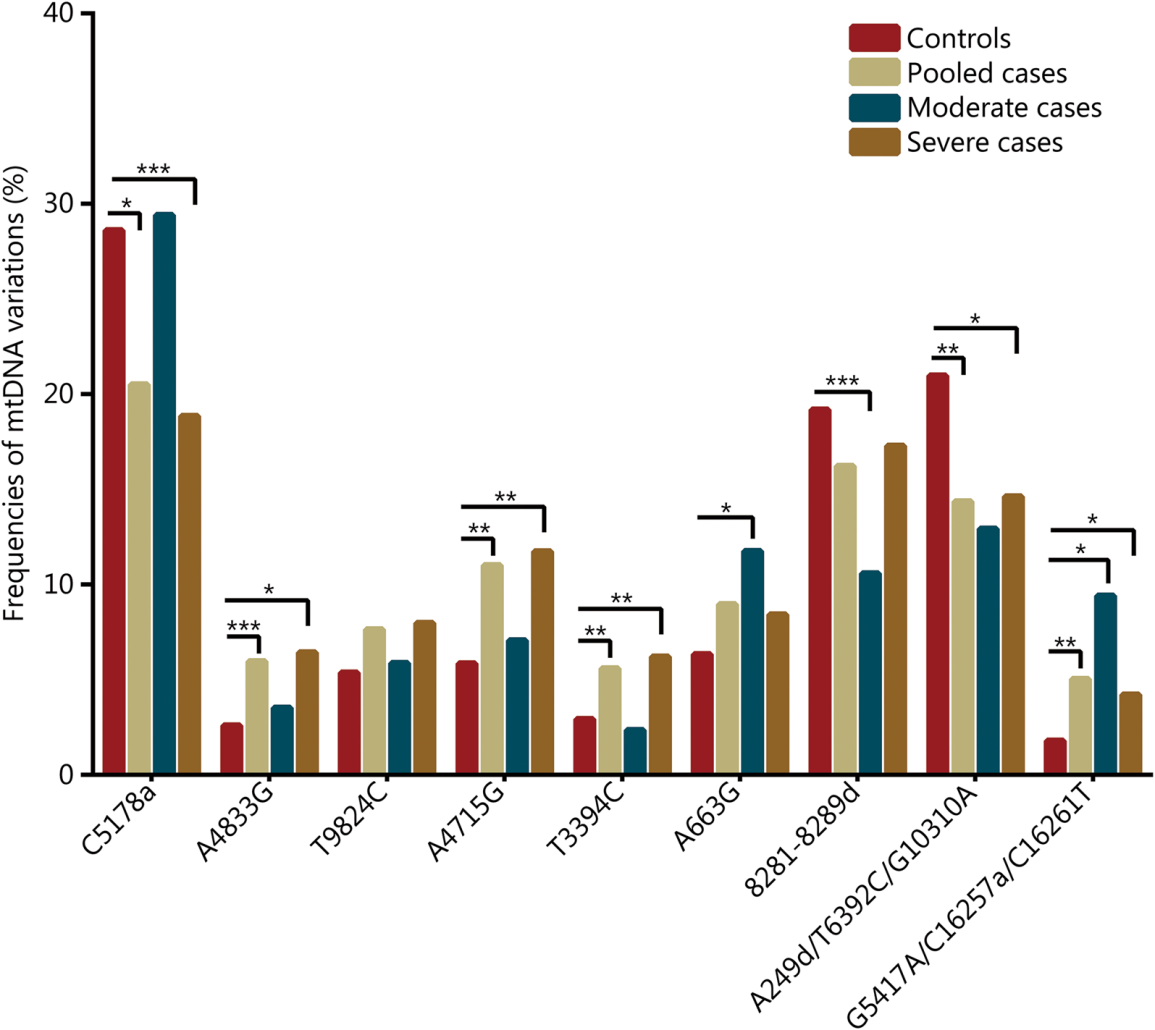
Frequencies of mtDNA variants defining mtDNA haplogroups in controls and COVID-19 patients. Significant differences between the two groups are labelled as follows: *P < 0.05, **P < 0.01, ***P < 0.001 [adjusted *P* value was determined by multivariate logistic regression analysis, adjusted for age, sex, smoking and diseases (including cardiopulmonary diseases, diabetes, obesity and hypertension)]

In total, 536 unrelated COVID-19 patients and 615 healthy controls were recruited in this study. As shown in Table 1, the COVID-19 patients smoked more cigarettes than controls (*P* < 0.001). After the cases were stratified into moderate and severe cases, the severe patients were found to be older than controls (*P* < 0.001). MtDNA variants defining mtDNA haplogroups were detected for all subjects. Pearson’s chi-squared test or Fisher’s exact test showed that mtDNA variants A4833G (*ND*2, defining mtDNA haplogroup G), A4715G (*ND*2, defining mtDNA haplogroup M8), T3394C (*ND1*, defining mtDNA haplogroup M9), and G5417A (*ND*2)/C16257a (D-loop)/C16261T (D-loop) (defining mtDNA haplogroup N9a) were significantly higher (*P* = 0.005, 0.002, 0.027 and 0.003, respectively), while mtDNA variants C5178a (*ND2*, defining haplogroup D; the letter “a” indicates nucleotide transversion) and A249d (D-loop)/T6392C (cytochrome c oxidase I gene, *CO1*)/G10310A (*ND*3) (defining haplogroup F; the letter “d” indicates nucleotide deletion) were significantly lower in COVID-19 patients than in controls (*P* = 0.002 and 0.004, respectively). When Bonferroni correction was applied, A4715G, G5417A/C16257a/C16261T and C5178a reached the required *P* value of < 0.0033 (0.05/15). Multivariate logistic regression analyses with adjustments for age, sex, smoking and diseases revealed that, based on a *P* value of < 0.05, A4833G, A4715G, T3394C and G5417A/C16257a/C16261T were associated with an increased risk of COVID-19 (*OR* = 4.384, 95% CI 2.103–9.137, *P* < 0.001; *OR* = 1.876, 95% CI 1.167–3.021, *P* = 0.009; *OR* = 2.618, 95% CI 1.339–5.128, *P* = 0.005; and *OR* = 3.401, 95% CI 1.486–7.752, *P* = 0.004, respectively). In contrast, C5178a and A249d (D-loop)/T6392C (*CO1*)/G10310A (*ND*3) variants were associated with a reduced risk of COVID-19 (*OR* = 0.681, 95% CI 0.504–0.919, *P* = 0.012; and *OR* = 0.639, 95% CI 0.483–0.847, *P* = 0.002, respectively) (Table 2 and Fig. 1).

**Table 1:** Clinical characteristics of the study population

Characteristic	Controls (*n* = 615)	COVID-19 patients						
		Pooled (*n* = 536)	*P* value	Moderate (*n* = 85)	*P* value	Severe (*n* = 451)	*P* value*	*P* value^#^
Gender [*n*(%)]			0.590^a^		0.559^a^		0.413^a^	0.280^a^
Male	356 (57.89)	319 (59.51)		46 (54.11)		273 (60.53)		
Female	259 (42.11)	217 (40.49)		39 (45.88)		178 (39.47)		
Age (years, $\overline{x}$ ± SD)	58.29 ± 13.87	60.83 ± 14.46	0.052^b^	59.96 ± 14.10	0.300^b^	61.68 ± 14.83	< 0.001^b^	0.324^b^
Age [*n* (%)]			0.689^a^		0.977^a^		0.458^a^	0.634^a^
≤ 40	48 (7.87)	46 (8.58)		6 (7.09)		40 (8.87)		
40–50	106 (17.24)	82 (15.30)		15 (17.65)		67 (14.86)		
50–60	112 (18.21)	91 (16.98)		17 (20.00)		74 (16.41)		
60–69	217 (35.28)	186 (34.70)		31 (36.47)		155 (34.37)		
> 70	132 (21.46)	131 (24.44)		16 (18.82)		115 (25.50)		
Pack-years of smoking [*n*(%)]^c^			0.098^a^		0.522^a^		0.858^a^	0.509^a^
0–20	543 (88.29)	455 (84.89)		70 (82.35)		385 (85.37)		
> 20	72 (11.71)	81 (15.11)		15 (17.65)		66 (14.63)		
Mean pack-years (year, $\overline{x}$ ± SD)	6.35 ± 10.86	9.27 ± 12.54	< 0.001^b^	8.76 ± 10.24	0.054^b^	10.87 ± 15.67	< 0.001^b^	0.233^b^

^a^*χ*^2^-test or Fisher's exact test

^b^*t*-test

^c^The median number of pack-years of cigarette smoking of the pooled COVID-19 patients and healthy controls was utilized as the cut-off point to stratify the subjects (1 pack-year = 20 cigarettes per day for 1 year)

*Severe cases versus controls

^#^Severe cases versus moderate cases

**Table 2:** Frequencies of mtDNA variants defining mtDNA haplogroups among controls and pooled cases

MtDNA variations	mtDNA haplogroups	Controls (*n* = 615) [*n*(%)]	Pooled cases (*n* = 536) [*n*(%)]	*P* value (*χ*^2^)^a^	Adjusted *P* value^b^	*OR* (95% CI)^b^
A663G	A	39 (6.34)	48 (8.96)	0.117	0.060	1.567 (0.980–2.500)
8281-8289d ^c^	B	118 (19.19)	87 (16.23)	0.217	0.150	0.781 (0.557–1.093)
C5178a ^c^	D	176 (28.62)	110 (20.52)	0.002	0.012	0.681 (0.504–0.919)
A249d/T6392C/G10310A	F (F1 + F3)	129 (20.98)	77 (14.37)	0.004	0.002	0.639 (0.483–0.847)
A4833G	G	16 (2.60)	32 (5.97)	0.005	< 0.001	4.384 (2.103–9.137)
T9824C	M7	33 (5.37)	41 (7.65)	0.119	0.519	1.181 (0.712–1.957)
A4715G	M8 (M8a + C + Z)	36 (5.85)	59 (11.01)	0.002	0.009	1.876 (1.167–3.021)
T3394C	M9	18 (2.93)	30 (5.60)	0.027	0.005	2.618 (1.339–5.128)
G16274A	M2	8 (1.30)	5 (0.93)	0.399	0.896	0.899 (0.180–4.484)
T16126C	M3	10 (1.63)	7 (1.30)	0.616	0.466	0.572 (0.128–2.567)
T16311C	M10	9 (1.46)	6 (1.12)	0.592	0.418	0.509 (0.099–2.610)
G6023A	M13	4 (0.65)	4 (0.75)	1.000	0.514	0.566 (0.102–3.137)
T489C/C10400T/T14783C/G15043A	M*	1 (0.16)	1 (0.19)	1.000	NA	
G5417A/C16257a/C16261T	N9a	11 (1.79)	27 (5.04)	0.003	0.004	3.401 (1.486–7.752)
T1391C/T16311C	R1	7 (1.14)	2 (0.37)	0.187	0.219	0.353 (0.067–1.859)

^a^*χ*^2^-test or Fisher's exact test. Bonferroni corrected *P* < 0.05/*n* (*n* = 15)

^b^*P* value and *OR*s (95% CIs) determined by multivariate logistic regression analysis, adjusted for age, gender, smoking and diseases (including cardiopulmonary diseases, diabetes, obesity and hypertension determined through clinical and radiographic examinations)

^c^The letter “d” indicates nucleotide deletion, the letter "a" indicates nucleotide transversion

*NA* not available

### Frequencies of mtDNA variants defining mtDNA haplogroups in controls and moderate cases

When COVID-19 patients were stratified into cases with moderate (non-ICU) and severe (ICU) disease (patients with ARDS, multiple organ dysfunction, or metabolic acidosis), Pearson’s chi-squared test or Fisher’s exact test showed that the mtDNA variants A663G (12S rRNA, defining mtDNA haplogroup A) and G5417A/C16257a/C16261T were significantly higher (*P* ≤ 0.001), while the 8281–8289d variant (specific to mtDNA haplogroup B, located in a non-coding region) was significantly lower in moderate COVID-19 patients than in controls (*P* < 0.001). All of these mtDNA variants (A663G, G5417A/C16257a/C16261T, and 8281–8289d) reached the required *P* value of < 0.0033 when Bonferroni correction was applied. Multivariate logistic regression analyses with adjustments for age, sex, smoking and diseases showed that 8281–8289d was associated with a reduced risk of moderate COVID-19 (*OR* = 0.034, 95% CI 0.016–0.068, *P* < 0.001), whereas A663G and G5417A/C16257a/C16261T variants were related to an increased risk of moderate COVID-19 (*OR* = 2.445, 95% CI 1.116–5.348, *P* = 0.026 and *OR* = 3.984, 95% CI 1.325–11.904, *P* = 0.014, respectively) (Table 3 and Fig. 1).

**Table 3:** Distribution of mtDNA variants defining mtDNA haplogroups among controls and moderate cases

MtDNA variations	MtDNA haplogroups	Controls (*n* = 615) [*n*(%)]	Moderate cases (*n* = 85)[*n*(%)]	*P* value (*χ*^2^)^a^	Adjusted *P* value^b^	*OR* (95% CI)^b^
A663G	A	39 (6.34)	10 (11.76)	< 0.001	0.026	2.445 (1.116–5.348)
8281–8289d ^c^	B	118 (19.19)	9 (10.59)	< 0.001	< 0.001	0.034 (0.016–0.068)
C5178a ^c^	D	176 (28.62)	25 (29.41)	0.898	0.362	1.289 (0.747–2.222)
A249d/T6392C/G10310A	F	129 (20.98)	11 (12.94)	0.110	0.092	1.969 (1.896–4.326)
A4833G	G	16 (2.60)	3 (3.53)	0.494	0.712	1.182 (0.486–2.875)
T9824C	M7	33 (5.37)	5 (5.88)	1.000	0.765	1.185 (0.389–3.610)
A4715G	M8 (M8a + C + Z)	36 (5.85)	6 (7.06)	0.808	0.910	1.449 (0.700–3.003)
T3394C	M9	18 (2.93)	2 (2.35)	1.000	0.903	0.910 (0.199–4.167)
T489C/C10400T/T14783C/G15043A	M* (M2 + M3 + M10 + M13 + M)	32 (5.20)	6 (7.06)	0.446	NA	
G5417A/C16257a/C16261T	N9a	11 (1.79)	8 (9.41)	0.001	0.014	3.984 (1.325–11.904)
T1391C/T16311C	R1	7 (1.14)	0	0.607	0.999	0

^a^*χ*^2^-test or Fisher's exact test. Bonferroni corrected *P* < 0.05/*n* (*n* = 15)

^b^Adjusted *P* value and *OR*s (95% CIs) determined by multivariate logistic regression analysis, adjusted for age, gender, smoking and diseases (including cardiopulmonary diseases, diabetes, obesity and hypertension determined through clinical and radiographic examinations)

^c^The letter “d” indicates nucleotide deletion, the letter “a” indicates nucleotide transversion

*NA* not available

### Distribution of mtDNA variants defining mtDNA haplogroups in controls and severe cases

Pearson’s chi-squared test or Fisher’s exact test demonstrated that the variants A4833G, A4715G, T3394C and G5417A/C16257a/C16261T were significantly higher (*P* = 0.003, 0.001, 0.010 and 0.023, respectively), while C5178a and A249d/T6392C/G10310A were significantly lower in severe COVID-19 patients than in controls (*P* < 0.001 and *P* = 0.008, respectively). When Bonferroni correction was applied, A4833G, A4715G and C5178a remained significant. Multivariate logistic regression analyses with adjustments for covariates showed that C5178a and A249d/T6392C/G10310A variants were associated with a reduced risk of severe COVID-19 (*OR* = 0.590, 95% CI 0.428–0.814, *P* = 0.001 and *OR* = 0.654, 95% CI 0.457–0.936, *P* = 0.020), while A4833G, A4715G, T3394C and G5417A/C16257a/C16261T variants were related to an increased risk of severe COVID-19 (*OR* = 2.336, 95% CI 1.179–4.608, *P* = 0.015; *OR* = 2.033, 95% CI 1.242–3.322, *P* = 0.005; *OR* = 3.040, 95% CI 1.522–6.061, *P* = 0.002; and *OR* = 2.890, 95% CI 1.199–6.993, *P* = 0.018, respectively) (Table 4 and Fig. 1).

**Table 4:** Distribution of mtDNA variants defining mtDNA haplogroups among controls and severe cases

MtDNA variations	MtDNA haplogroups	Controls (*n* = 615) [*n*(%)]	Severe cases (*n* = 451) [*n*(%)]	*P* value (*χ*^2^)^a^	Adjusted *P* value^b^	*OR* (95% CI) ^b^
A663G	A	39 (6.34)	38 (8.43)	0.231	0.141	1.451 (0.884–2.381)
8281–8289d ^c^	B	118 (19.19)	78 (17.29)	0.472	0.202	0.796 (0.561–1.130)
C5178a ^c^	D	176 (28.62)	85 (18.85)	< 0.001	0.001	0.590 (0.428–0.814)
A249d/T6392C/G10310A	F	129 (20.98)	66 (14.63)	0.008	0.020	0.654 (0.457–0.936)
A4833G	G	16 (2.60)	29 (6.43)	0.003	0.015	2.336 (1.179–4.608)
T9824C	M7	33 (5.37)	36 (7.98)	0.101	0.390	0.795 (0.472–1.341)
A4715G	M8 (M8a + C + Z)	36 (5.85)	53 (11.75)	0.001	0.005	2.033 (1.242–3.322)
T3394C	M9	18 (2.93)	28 (6.21)	0.010	0.002	3.040 (1.522–6.061)
T489C/C10400T/T14783C/G15043A	M* (M2 + M10 + M11 + M13a + M)	32 (5.20)	17 (3.77)	NA		
G5417A/C16257a/C16261T	N9a	11 (1.79)	19 (4.21)	0.023	0.018	2.890 (1.199–6.993)
T1391C/T16311C	R1	7 (1.14)	2 (0.44)	0.316	0.358	0.460 (0.088–2.410)

^a^*χ*^2^-test or Fisher's exact test. Bonferroni corrected *P* < 0.05/*n* (*n* = 15)

^b^Adjusted *P* value and *OR*s (95% CIs) determined by multivariate logistic regression analysis, adjusted for age, gender, smoking and diseases (including cardiopulmonary diseases, diabetes, obesity and hypertension determined through clinical and radiographic examinations)

^c^The letter “d” indicates nucleotide deletion, the letter “a” indicates nucleotide transversion

*NA* not available

## Discussion

To provide insight into COVID-19 risk in terms of mtDNA variation, we conducted a case–control study in a Han Chinese population from Central China. Our data demonstrated that the most common investigated mtDNA variants (> 10% in the control population) at C5178a (*ND*2) and A249d (D-loop)/T6392C (*CO1*)/G10310A (*ND3*) contributed to an individual’s resistance to developing severe COVID-19, whereas A4833G (*ND2*), A4715G (*ND2*), T3394C (*ND1*) and G5417A (*ND2*)/C16257a (D-loop)/C16261T (D-loop) polymorphisms increased the risk of severe COVID-19 in this population. Additionally, the mtDNA variants A663G (12S rRNA) and G5417A/C16257a/C16261T increased an individual’s risk of developing moderate COVID-19, while the 8281–8289d variant (located in a non-coding region) decreased an individual’s risk of developing moderate COVID-19. The mtDNA variants G5417A/C16257a/C16261T were risk factors for both moderate and severe COVID-19.

As the hub of cellular oxidative homeostasis, mitochondria not only play a role in oxidative stress and inflammation in SARS-CoV-2 infection and COVID-19 development [8, 9, 21–24], but are also indispensable regulators of the innate and adaptive immune responses in the process of SARS-CoV-2 infection and COVID-19 development [10, 11, 24–26]. Moreover, mitochondrial residency of SARS-CoV-2 with a stronger signal compared to its coronavirus relatives further implied that mitochondria are the major cellular organelle affected by oxidative stress and inflammation caused by SARS-CoV-2 infection [27]. In comparison with nuclear DNA, mtDNA is particularly susceptible to oxidative damage due to its direct exposure to ROS, limited DNA repair capacity and absence of protection by histones. The decline in mitochondrial function with aging might explain the phenomena of high mortality rate in elderly COVID-19 patients to a certain extent [1, 13, 17]. Thus, when SARS-CoV-2 infects cells, the common “nonpathological” mtDNA variants, which define mtDNA haplogroups and determine OXPHOS performance and ROS production, contribute to an individual’s capacity for antioxidant and immune responses to protect cells from SARS-CoV-2 infection and COVID-19 development or can aggravate the process. Consistent with this, the mtDNA variation C5178a (Leu237Met) in *ND2*, defining mtDNA haplogroup D and proposed to be an efficient oxidant scavenger [28], was significantly lower in both the total cohort of COVID-19 patients and severe COVID-19 patients compared to controls in this study. The protective effect of the C5178a mtDNA variant has been reported to increase human longevity [13], to be beneficial for diabetic patients against atherosclerotic and myocardial infarction [29], and to decrease an individual’s risk of developing acute mountain sickness, lung cancer, chronic obstructive pulmonary disease (COPD) and other diseases [16–18, 29]. Therefore, the protective effect of C5178a against oxidative damage as an efficient oxidant scavenger might protect cells from the oxidative destruction caused by SARS-CoV-2 infection and decrease an individual’s risk of developing COVID-19, especially for severe COVID-19.

The A249d (D-loop), T6392C (*CO1*, synonymous mutation) and G10310A (*ND*3, synonymous mutation) variants are common variants of mtDNA sub-haplogroups F1–4 [30]. In our study, G12406A (Val24Ile in *ND*5, defining mtDNA sub-haplogroup F1) and T16298C/C16304T/T16362C (D-loop, defining mtDNA sub-haplogroup F3) were detected, and both had significantly lower frequencies in COVID-19 patients (*P* = 0.043 and 0.018, respectively). As expected, the combined sub-haplogroups F1 and F3 (representing haplogroup F) were associated with a decreased risk of COVID-19 and severe COVID-19. In Asian populations, haplogroup F is a positive factor associated with a long life-span [31], confers beneficial effects on the resistance of metabolic syndrome (MetS) [32], and improves the physical performance of athletes [33]. A249d occurs within the H-strand replication origin and mitochondrial transcription factor A (mtTF1) binding site, suggesting that A249d may have an impact on mtDNA replication and transcription. Recently, synonymous mutations have been reported to alter translation speed through codon optimality and protein folding in the nuclear genome, which may impact cell fitness [34]. Thus, we deduced that the variants A249d (D-loop)/T6392C (*CO1*)/G10310A (*ND3*) might have protective functions against COVID-19 by regulating the replication and transcription of mtDNA and/or the translation speed through codon optimization, protein folding or other mechanisms.

The mtDNA variants A4833G (Thr122Ala in NADH dehydrogenase subunit 2, *ND2*), A4715G (synonymous mutation in *ND2*), T3394C (Tyr30His in *ND1*) and G5417A (synonymous mutation in *ND2*)/C16257a (D-loop)/C16261T (D-loop) were found to increase an individual’s risk of developing severe COVID-19 in our study. Of note, A4715G, T3394C and G5417A/C16257a/C16261T are reported to be associated with an increased risk of type II diabetes mellitus (T2DM) in the Chinese population [35]. A4715G is a risk factor for moderate and severe non-alcoholic fatty liver disease [36]. Meanwhile, T3394C helps native Tibetans adapt to hypoxic environments because it has higher complex I activity [15]. However, in low-altitude areas, T3394C increases an individual’s risk of many diseases, including Leber’s hereditary optic neuropathy [37], hypertension [38] and T2DM [39]. Additionally, T3394C is a candidate variant that counteracts longevity [40]. Similar to T3394C, A4833G is significantly more frequent in native Tibetans residing at high altitudes than in Han Chinese individuals living in low-altitude areas (32/289 vs. 39/1605, *P* = 0.0001) [15], which might help native Tibetans adapt to hypoxic environments. Similarly, A4833G is a risk factor for lung cancer [18], COPD [19] and recurrent oral ulceration in low-altitude areas [41]. Therefore, T3394C and A4833G might be risk factors for severe COVID-19 through the same mechanism as in other human diseases occurring in the low-altitude areas.

The mtDNA variant G5417A in *ND2* (synonymous mutation, specific for mtDNA haplogroup N9) confers a higher risk of MetS development in HIV-infected patients [42]. In the Chinese population, G5417A/C16257a/C16261T is a risk factor for diabetic nephropathy due to more ROS and fragmented mitochondria [35], which might account for it being a risk factor for both moderate and severe COVID-19 in this Han Chinese population.

Interestingly, certain variants in human mtDNA significantly change specific OXPHOS enzyme activity and response to OXPHOS targeting agents in yeast models [20], indicating that the mtDNA variants detected in this study might alter COVID-19 severity by affecting specific OXPHOS enzyme activity and the response to certain drugs/treatments. Therapeutic regimens based on the variant status of mtDNA may improve the outcomes of patients with COVID-19.

Of note, a mtDNA deletion of approximately 800 bp was detected during the PCR–RFLP analysis in this study. In our previous studies, an 822 bp mtDNA deletion was identified and demonstrated to be positively associated with cigarette smoking and mtDNA haplogroups [18, 19]. Because the blood samples used in this study were incubated at 55 °C for 30 min to inactivate any potential SARS-CoV-2, the mtDNA deletion was not further analysed to avoid possible mtDNA breakage caused by incubation at a higher temperature.

This study had some limitations. First, during the extreme clinical circumstances of the pandemic, especially at the beginning of the epidemic, we were unable to recruit asymptomatic patients and collect detailed clinical data (for example, levels of inflammatory cytokines, immune factors and disease outcome) in a very short period of time, which will be important to investigate in follow-up studies. Second, PCR–RFLP and HVS-I sequencing approaches were used to identify mtDNA variants in the study. Although they can detect known variants defining mtDNA haplogroups, these methods are unable to detect other mtDNA variants and heteroplasmy information on all variants, as other novel techniques such as the next generation sequencing (NGS) of mtDNA do. In future projects, NGS and other novel methods will be utilized to provide more comprehensive information on mtDNA variation. Third, two mtDNA macrohaplogroups M and N, emerging from African-specific mtDNA L3 in Northeast Africa, left Africa successfully and colonized the rest of the world. The mtDNA macrohaplogroup N gave rise to multiple European, Asian and Native American mtDNA lineages (including N1, N2, N9, N*, A, X, R0, JT, R9, R*, B and U, while mtDNA macrohaplogroup M gave rise to only Asian and Native American haplogroups (consisting of M7, M8, M9, G, D and M*). Of all the Asian mtDNA lineages, only haplogroups A, C, D and X became enriched in Northeast Siberia and crossed the Bering Land Bridge into the Americas and haplogroup B colonized the Pacific Islands, demonstrating that haplogroups A, C, D and X have been subjected to much greater cold stress than haplogroup B or the African macro-haplogroup L [43, 44]. These findings show that human mtDNA exhibits dramatic and region-specific sequence variation in geographically localized indigenous populations. In China, with economic development and population flow, the frequencies of mtDNA variations defining mtDNA haplogroups vary across populations. Because we only analysed mtDNA variations in the Han Chinese population from Central China, large-scale studies are needed in other populations.

## Conclusions

Our findings revealed for the first time that the common mtDNA variants at C5178a (*ND2*) and A249d (D-loop)/T6392C (*CO1*)/G10310A (*ND3*) contributed to resistance to COVID-19 development, whereas A4833G (*ND2*), A4715G (*ND2*), T3394C (*ND1*) and G5417A (*ND2*)/C16257a (D-loop)/C16261T (D-loop) variants may be risk factors, providing evidence that the gene-environment interactions exist in COVID-19 progression.

## Supplementary Information

**Additional file 1: Table S1**. The primers for PCR-RFLPs and HVS-I sequencing. **Table S2**. The Human Genome Variation Society (HGVS) validation of mtDNA variations mentioned in the study

## Data Availability

The data that supported the findings of this study are available from the corresponding author upon reasonable request.

## Abbreviations

ARDS: Acute respiratory distress syndrome
CIs: Confidence intervals
CO1: Cytochrome c oxidase I
COVID-19: Coronavirus disease 2019
COPD: Chronic obstructive pulmonary disease
HGVS: The Human Genome Variation Society
HVS-I: Hypervariable segment I
HVS-II: Hypervariable segment II
mtDNA: Mitochondrial DNA
MetS: Metabolic syndrome
ND1: NADH dehydrogenase subunit 1
ND2: NADH dehydrogenase subunit 2
ND5: NADH dehydrogenase subunit 5
NGS: The next generation sequencing
OXPHOS: Oxidative phosphorylation
ORs: Odds ratios
PCR–RFLP: PCR-restriction fragment length polymorphism
ROS: Reactive oxygen species
SARS-CoV-2: Severe acute respiratory syndrome coronavirus 2
T2DM: Type II diabetes mellitus
