# Supplementary material for: Common mtDNA variations at C5178a and A249d/T6392C/G10310A decrease the risk of severe COVID-19 in a Han Chinese population from Central China
Source: Mil Med Res. 2021 Nov 1;8:57. doi: 10.1186/s40779-021-00351-2 (PMC8558762; doi:10.1186/s40779-021-00351-2)
Supplement: Supplementary file 1 — Additional file 1: Table S1. The primers for PCR-RFLPs and HVS-I sequencing. Table S2. The Human Genome Variation Society (HGVS) validation of mtDNA variations mentioned in the study [file 40779_2021_351_MOESM1_ESM.docx]

**Table S1** Primers used for PCR-RFLPs and HVS-I sequencing

| **Primer number** | | **Sequences (5’-3’)** |
| --- | --- | --- |
| Primer 1 | Forward | AACCAAACCCCAAAGACACC |
|  | Reverse | ATCCACCTTCGACCCTTAAG |
| Primer 2 | Forward | GTAAGCGCAAGTACCCACG |
|  | Reverse | ATTGGTGGCTGCTTTTAGGC |
| Primer 3 | Forward | AATTTGCCCACAGAACCCTC |
|  | Reverse | GACTCTAGAATAGGATTGCGC |
| Primer 4 | Forward | GTCCTAAACTACCAAACCTGC |
|  | Reverse | ATGATGGCTAGGGTGACTTC |
| Primer 5 | Forward | CGCTCTTCTACTATGAACCC |
|  | Reverse | TGTGCCTGCAAAGATGGTAG |
| Primer 6 | Forward | ATAATAGGAGCTTAAACCCCC |
|  | Reverse | GTGTTAGTCATGTTAGCTTG |
| Primer 7 | Forward | AGCAGTTCTACCGTACAACC |
|  | Reverse | TAAGGAGGCTTAGAGCTGTG |
| Primer 8 | Forward | GAAAATCACCTCGGAGCTGG |
|  | Reverse | TGTGCTCACACGATAAACCC |
| Primer 9 | Forward | ACCTCAACACCACCTTCTTC |
|  | Reverse | TTTGAAAAAGTCATGGAGGCC |
| Primer 10 | Forward | GATTTGAGAAGCCTTCGCTTC |
|  | Reverse | GCTATAGGGTAAATACGGGC |
| Primer 11 | Forward | TCTTGCACTCATGAGCTGTC |
|  | Reverse | CCAATTAGGTGCATGAGTAGG |
| Primer 12 | Forward | CTCATTTACACCAACCACCC |
|  | Reverse | GCCAATAATGACGTGAAGTCC |
| Primer 13 | Forward | TCCCACTCCTAAACACATCC |
|  | Reverse | GTTGAGGGTTATGAGAGTAGC |
| Primer 14 | Forward | TCTGGCCTATGAGTGACTAC |
|  | Reverse | AAGTGGAGTCCGTAAAGAGG |
| Primer 15 | Forward | TTCACAGCCACAGAACTAATC |
|  | Reverse | AAACCCGGTAATGATGTCGG |
| Primer 16 | Forward | GCCCACGGGCTTACATC |
|  | Reverse | AAACCGATATCGCCGATACG |
| Primer 17 | Forward | TCGTTACATGGTCCATCATAG |
|  | Reverse | AAGCGAGGTTGACCTGTTAG |
| Primer 18 | Forward | TCCATCATCCACAACCTTAAC |
|  | Reverse | GATTGTTAGCGGTGTGGTCG |
| Primer 19 | Forward | TCTTCCCACTCATCCTAACC |
|  | Reverse | CCCCTCAGAATGATATTTGGC |
| Primer 20 | Forward | TCCAACATCTCCGCATGATG |
|  | Reverse | TCTCCGGTTTACAAGACTGG |
| Primer 21 | Forward | TTCGCCTACACAATTCTCCG |
|  | Reverse | ACAGATACTGCGACATAGGG |
| Primer 22 | Forward | GTCAAATCCCTTCTCGTCCC |
|  | Reverse | TTTATGGGGTGATGTGAGCC |

**Table S2** Human Genome Variation Society (HGVS) validation of mtDNA variations mentioned in the study

| **MtDNA variations** | Constructed HGVS variant description | **Genomic description** | **Created** | **Deleted** |
| --- | --- | --- | --- | --- |
| 249A>d | NC_012920.1:m.[249A>d;>] | NC_012920.1:m.249del |  |  |
| 489T>C | NC_012920.1:m.489T>C | NC_012920.1:m.489T>C |  |  |
| 663A>G | NC_012920.1:m.[663A>G;>] | NC_012920.1:m.663A>G | BstNI, HaeIII, LpnPI (2), PspGI, ScrFI, StyD4I |  |
| 1391T>C | NC_012920.1:m.1391T>C | NC_012920.1:m.1391T>C |  |  |
| 3394T>C | NC_012920.1:m.[3394T>C;>] | NC_012920.1:m.3394T>C |  |  |
| 4715A>G | NC_012920.1:m.4715A>G | NC_012920.1:m.4715A>G | BsrDI, NciI, ScrFI, StyD4I |  |
| 4833A>G | NC_012920.1:m.[4833A>G;>] | NC_012920.1:m.4833A>G | BsaHI, HaeII, HhaI, HinP1I, KasI, NarI, PluTI, SfoI |  |
| 5178C>a | NC_012920.1:m.5178C>A | NC_012920.1:m.5178C>A |  | AluI, CviKI_1 |
| 5417G>A | NC_012920.1:m.5417G>A | NC_012920.1:m.5417G>A | MluCI | HpyCH4III |
| 6023G>A | NC_012920.1:m.6023G>A | NC_012920.1:m.6023G>A | BmrI, BsrI | AluI, BseYI, NmeAIII |
| 6392T>C | NC_012920.1:m.6392T>C | NC_012920.1:m.6392T>C |  |  |
| 8281-8289d | NC_012920.1:m.8281_8289del | NC_012920.1:m.8281_8289del |  |  |
| 9824T>C | NC_012920.1:m.[9824T>C;>] | NC_012920.1:m.9824T>C | HinfI, MlyI, PleI, XcmI |  |
| 10310G>A | NC_012920.1:m.[10310G>A;>] | NC_012920.1:m.10310G>A |  | BfuAI, BspMI, LpnPI |
| 10400C>T | NC_012920.1:m.[10400C>T;>] | NC_012920.1:m.10400C>T |  |  |
| 14783T>C | NC_012920.1:m.[14783T>C;>] | NC_012920.1:m.14783T>C |  | AseI, PacI |
| 15043G>A | NC_012920.1:m.[15043G>A;>] | NC_012920.1:m.15043G>A | Hpy188I |  |
| 16126T>C | NC_012920.1:m.[16126T>C;>] | NC_012920.1:m.16126T>C | HpyCH4V |  |
| 16257C>a | NC_012920.1:m.[16257C>A;>] | NC_012920.1:m.16257C>A |  | CviKI_1 |
| 16261C>T | NC_012920.1:m.[16261C>T;>] | NC_012920.1:m.16261C>T |  |  |
| 16274G>A | NC_012920.1:m.[16274G>A;>] | NC_012920.1:m.16274G>A |  | BciVI |
| 16311T>C | NC_012920.1:m.[16311T>C;>] | NC_012920.1:m.16311T>C | MwoI |  |
